# Emotional Arousal During Social Stress in Young Adults With Autism: Insights From Heart Rate, Heart Rate Variability and Self-Report

**DOI:** 10.1007/s10803-019-04000-5

**Published:** 2019-04-04

**Authors:** Renee R. Dijkhuis, Tim Ziermans, Sophie van Rijn, Wouter Staal, Hanna Swaab

**Affiliations:** 1 Present Address: Department of Clinical Child and Adolescent Studies, Neuropedagogics and Developmental Disorders, Leiden University, Pieter de la Court Building, Wassenaarseweg 52, 2333 AK Leiden, The Netherlands; 2 Leiden Institute for Brain and Cognition, Leiden, The Netherlands; 3 Department of Psychology, University of Amsterdam, Amsterdam, The Netherlands; 4 Karakter Universitair Centrum, Nijmegen, The Netherlands; 5 Radboud Universitair Medisch Centrum, Nijmegen, The Netherlands; 6 Present Address: Department of Brain and Cognition, University of Amsterdam, Amsterdam, The Netherlands

**Keywords:** Physiological arousal, Emotion regulation, Young adulthood, Social stress, Emotional awareness

## Abstract

In order to better understand which factors play a role in non-adaptive social behavior in autism spectrum disorder (ASD) we looked into physiological arousal and awareness of one’s own emotions. Heart rate (HR) and heart rate variability (HRV) were measured during a public speaking task in 51 young adults with ASD and 28 typically developing (TD) controls. The results showed no significant group differences in baseline HR/HRV, HR reactivity (change from baseline to the speaking task) or self-reported emotional awareness. However, adults with ASD showed significantly lower HRV reactivity (*p* = .023, *d* = 0.6) compared to TD adults. These results suggest a mismatch between arousal regulation and emotional awareness, which may be related to problems in social adaptation in ASD.

**Electronic supplementary material:**

The online version of this article (10.1007/s10803-019-04000-5) contains supplementary material, which is available to authorized users.

Autism spectrum disorder (ASD) is a complex condition with impairments in socio-emotional functions, stereotyped behavior and atypical sensory behaviors. The problems many individuals with ASD encounter in social functioning have been related to arousal, attention abnormalities and deficits in emotion regulation (Mazefsky et al. 2013). Emotion regulation is a complex and multifaceted construct that involves physiological, behavioral and cognitive processes, which allow an individual to monitor, evaluate, and modify emotional reactions to accomplish one’s goals (Adrian et al. 2011; Thompson et al. 2008) and the number of studies showing that children with ASD have difficulties regulating their emotions has accelerated over the past few years (Bachevalier and Loveland 2006; Guy et al. 2014; Loveland 2005; Mazefsky et al. 2013; Samson et al. 2015; Zantinge et al. 2017). Being able to accurately label negative emotional states is considered a fundamental prerequisite for adequate coping with the emotional consequences of a social situation and for managing associated behavioral impulses, leading to better social adaptation (Barrett et al. 2001).

Even before an individual (un)consciously acts to control his/her emotions, internal regulatory processes take place at a physiological level. When perceiving a stressor, for example a critical evaluation by an audience, self-regulating processes start by automatically activating the autonomic nervous system (ANS) (Sapolsky 1998). Within the ANS, beat-to-beat heart rate (HR) and the variation in time between heartbeats, also known as cardiac control or heart rate variability (HRV), plays an important role. Previous studies have suggested that HRV is related to socio-emotional behaviors and skills like social responsiveness, emotion recognition, peer engagement and spontaneous eye gaze in both children with- and without autism (Bal et al. 2009; Eisenberg et al. 2010; Fabes et al. 1993; Heilman et al. 2008; Henderson et al., 2004; Patriquin et al. 2014; Patriquin et al. 2011). Two commonly cited models argue for the importance of HRV in relation to socio-emotional behaviors are: (1) The Polyvagal Theory (e.g. Porges 2001, 2007; Porges et al. 2013) asserts that individual differences in HRV (an index for vagal tone as measured with respiratory sinus arrhythmia; RSA) mediate emotion regulation and expression; and (2) The Neurovisceral Integration Model (e.g. Thayer and Lane 2000; Thayer et al. 2012) posits that a core set of neutral structures integrates signals from inside and outside the body to assess whether the physiological state of the body, cognition, perception and action match the environmental demands. HRV may therefore be a good marker for investigating behavior in socially changing environments in individuals with ASD. In their paper, Thayer and Lane (2000) argue that people with low HRV are less able to detect and experience safety, even if it is present, and experience difficulties with affective information processing.

Many studies have reported on physiological arousal in children with ASD and results are suggestive of normal HR during rest compared to typically developing (TD) controls (Benevides and Lane 2015), but lower HRV (Bal et al. 2009; Klusek et al. 2014; Ming et al. 2005; Neuhaus et al. 2014; Porges et al. 2013; Van Hecke et al. 2009). The change in response from rest (tonic) activity to the task-engaged phases (phasic) is indicated by reactivity and reflects the alertness of the body to the demands of the environment. Studies with children and adolescents with ASD have shown less HRV reactivity to challenging tasks compared to TD controls (Althaus et al. 1999; Toichi and Kamio 2003a; Van Hecke et al. 2009). An example of a social task that triggers high arousal levels is the Trier Social Stress Test (TSST; (Kirschbaum et al. 1993). The TSST consists of a presentation task and serial subtractions in front of a jury with the goal of inducing evaluative stress. The advantage of using the TSST is that it is a laboratory-controlled assessment evoking negative emotions through stressful tasks in a social-evaluative situation, allowing for experimental induction of emotional arousal and the observation of reactivity from baseline to challenging conditions. In a meta-analysis (Shahrestani et al. 2015) of studies reporting on HRV during social interaction tasks like the TSST in adolescents and adults with- and without psychopathology, it was found that TD participants showed reduced HRV from baseline to stressful tasks in the TSST, while those with psychopathology did not. So far, studies in youth with ASD using the TSST show contradicting results. Edmiston et al. (2016) found that male adolescents with ASD showed less change in RSA throughout the TSST compared to their TD peers while others have reported no differences in HR or HRV reactivity between children and adolescents with ASD and TD controls (Levine et al. 2012; Mertens et al. 2017). However, the ASD groups were small (n ≤ 20) in these studies. In a study by Hollocks et al. (2014) 20 ASD subjects, 32 ASD subjects with a comorbid anxiety disorder and 23 controls were evaluated. No group differences were detected in any of the HRV measurements during the TSST, but a blunted HR and cortisol response to the psychosocial stress was detected in the ASD group. As the ANS is known to mature through childhood, with older children showing better regulation of emotion than infants (Porges et al. 1994) and stability of physical activity is lower in early childhood than in adulthood (Telama 2009), it would be valuable to see if these findings can be replicated in adults with ASD.

Only few studies have been published on arousal in adults with ASD, which have found comparable HR and HRV during rest (Smeekens et al. 2013; Toichi and Kamio 2003b) and in response to social tasks (Smeekens et al. 2013), compared to TD peers. In studies using the TSST, blunted HR reactivity has been found in adults with ASD (Jansen et al. 2006; Smeekens et al. 2013) compared to adults without ASD, indicating a decreased physiological response to the stressful phase. However, to our knowledge, HRV reactivity has not been reported in adults with ASD and none of the studies mentioned above included a simultaneous self-report measure for emotional awareness, which could provide more insight into the conscious processing of physiological arousal in ASD.

A few existing studies have addressed emotional awareness of arousal in ASD without physiological measures. In a study by Bölte et al. (2008), a small group of adults with autism reported higher levels of experienced arousal when viewing neutral pictures and lower arousal when viewing sad stimuli compared to TD controls, which is suggestive of deviant awareness of emotional arousal in ASD. In line with this notion, studies looking at self-reports of individuals with ASD have found that they are generally less able to identify and describe their own emotions (Dijkhuis et al. 2017; Hill et al. 2004; Samson et al. 2012; Ziermans et al. 2018). It has also been shown that, compared to TD individuals, adults with ASD report higher levels of negative emotions but similar levels of positive emotions (Samson et al. 2012) and in children and adolescents with ASD emotion dysregulation appears to be related to all core features of the disorder (Samson et al. 2014).

The current study aimed to further address the contribution of emotional arousal regulation to social (dys) functioning in young adults with ASD. Physiological measures were included in a social stress paradigm to investigate if young adults with ASD would display deviating levels of arousal regulation compared to their TD peers. Specifically, it was hypothesized that individuals with ASD would show less HR and HRV reactivity in response to social stress than TD individuals, since reactivity from baseline to stress is considered to be a marker for adaptation to changing environments. Also, based on previous research, we expected comparable HR during baseline and stressful tasks, but lower baseline HRV in the ASD group compared to TD controls. In addition, it was expected that ASD individuals would show less self-reported emotional awareness after task completion than their TD peers.

## Methods

### Participants

Fifty-one young adults with ASD (*M*_age_ = 22.48, *SD* = 2.43) and 28 TD peers (*M*_age_ = 20.65, *SD* = 1.57) were recruited for this study, which is part of a study project on study progress and quality of life in students with ASD within the higher education system. All participants were postsecondary students enrolled in higher education in the Netherlands. To increase generalizability, both males and females were included in the study and the groups were matched on sex (both 72% male). The ASD group was recruited through Stumass; a non-profit organization providing group home accommodation and services for students with ASD who are enrolled in university programs or universities of higher professional education. The goal of Stumass is to support students towards independency in personal, social and academic life. In order to be enrolled in Stumass, applicants are required to have received a recent formal clinical diagnosis of ASD based on the Diagnostic Statistic Manual of Mental Disorders (DSM) criteria (version dependent on what was customary at the time of referral), provided according to Dutch protocol. An additional requirement for enrollment in Stumass is that psychiatric co-morbidity, if present at entry, is either in remission or of minimal impact on daily functioning of the student.

For the control group, college students from the city of Leiden and neighboring regions were recruited through information brochures and an online student recruitment platform at Leiden University. Extra emphasis was placed on recruiting across different higher education faculties to better match the academic profile of the ASD group. Participants who reported having received a DSM diagnosis during their lifetimes, were not included in the control group. In order to screen for global level of autism symptom severity, the Dutch self-report version of the Social Responsiveness Scale for Adults (SRS-A; Constantino and Todd 2005) was administered to all participants. An additional exclusion criterion for both groups was an Intelligence Quotient (IQ) below 80, which was checked by the V-BD short form. Total IQ was estimated based on a long-standing method in the short-form literature with the formula [3 × (sum of normed scores) + 40] (Tellegen and Briggs 1967). The V-BD short form correlates highly with the estimated Full Scale Intelligence Quotient (TIQ) of the WAIS-IV (r = 0.86) (Denney et al. 2015) and is considered a valid estimation of intelligence, with good reliability and validity in both clinical (Denney et al. 2015; Girard et al. 2015) and non-clinical populations (Crawford et al. 2008). For their participation in the study all participants were rewarded with a voucher of 20 Euros. The research protocol was approved by the Medical Ethics Committee of Leiden University Medical Center and written informed consent was obtained from all participants before participation.

At the time of assessment nineteen participants in the ASD group were on (multiple) prescribed medications, including medications that are known to influence ANS functioning, see Table 1 for a complete overview. TD participants that reported current psychotropic medication use (*n* = 2) were removed for further analyses. As medication effects on physiological arousal have generated conflicting results in ASD and the literature is inconclusive about reliable attenuation or elevation of situational arousal (Mertens et al. 2017), it was decided not to exclude participants based on medication use but to control statistically for the influence of medication with a (potential) registered effect on HR and HRV.

**Table 1:** Medication use

Type of medication	Active ingredient	Number of participants	
		ASD	TD
Stimulants	Dexamphetamine	2	
	Methylphenidate	6	1
Antipsychotics	Aripiprazole	1	
	Quetiapine	1	
	Risperidone	3	
SSRI	Citalopram	5	
	Escitalopram	1	
	Paroxetine		1
	Sertraline	1	
	Fluoxetine	1	
Other	Bupropion	1	

### Social Stress Task

**Fig. 1:**
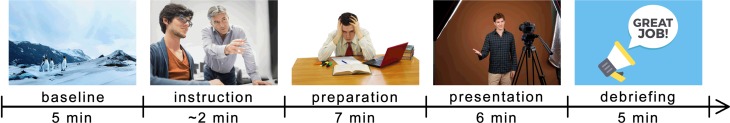
The social stress task paradigm

The task used in this study is based on the public speaking paradigm of the TSST, which is often used to measure ANS regulation during stress and has been found to provoke the most robust arousal responses when compared with other stress tasks (Dickerson and Kemeny 2004). The task was adapted in a manner to create a “real-life” scenario for higher education students, as they are required to give presentations and talks in front of their peers and increasingly via online platforms throughout higher education. The paradigm consisted of the following phases (see Fig. 1): (1) a neutral video of a nature scene without sound (baseline measure; 5 min), (2) explanation of the upcoming presentation phase by the experimenter, (3) preparation phase for delivering a personal presentation (7 min), (4) presentation in front of a jury member via webcam (6 min), and (5) a questionnaire concerning emotional awareness and verbal debriefing.

One of the investigators was present in the room—outside of view of the participant—behind a laptop to insert the markers for synchronizing eye tracking and arousal data, and another was the designated instructor adjacent to the participant. After the baseline measure, the instructor informed participants that they had 7 min to prepare a story about themselves and would present their story in English to an international jury member via a live webcam connection. The instructor told the participants that the jury member was instructed not to speak to the participant during the presentation. In reality participants were shown a pre-recorded video clip of an actor staring directly into the camera who showed minimal movement and did not talk, leading the participant to question the accuracy of their own behavior and presumably facilitating negative emotional arousal. After the preparation phase, the instructor set up a video camera and informed the participant that their performance would be recorded, evaluated and compared to other participants in the study. The investigator behind the laptop made a sham online connection with the jury member and the participants were prompted to start their presentation once the video appeared. After 6 min, the actor in the video made a stop signal with her hands to indicate the end of the presentation phase. If the participant stopped talking prior to the 6-min mark, the experimenter verbally encouraged the participant to continue talking. The participants remained seated behind the laptop throughout the entire task. Additionally, the experimenter assisted if necessary with the questionnaires in between videos to ensure minimal movement.

### Visual Attention

To monitor attention directed towards the social stressor (the jury member during the presentation phase) and to be able to control for any group differences in focus on the social stressor, eye tracking was used to measure gaze behavior. The videos of the Social Stress Task (for the baseline and the presentation phase) were displayed on a 15.6-in. LCD screen and gaze data was processed by the Tobii T120 Eye Tracker (Tobii Technology, Sweden). Gaze behavior during the presentation phase was measured with the Tobii eye tracker, which records the X and Y coordinates of participant’s eye position by using corneal reflection techniques. Gaze fixations and pupil responses were sampled at a frequency of 120 Hz. Gaze data were processed in Tobii Studio (3.2.1) using the Tobii I-VT filter. A dynamic AOI of the face of the jury member was defined in Tobii studio with the ‘Dynamic AOI (Area of Interest)’ tool, using freeform and ellipse shapes. The face AOI was defined with 1 cm margin; large enough to reliably capture the gaze fixation (Hessels et al. 2015). In Tobii studio, visit duration at the whole screen and fixation duration at the face AOI was calculated for the presentation task. Visit duration represents the time spent gazing on a specific AOI while fixation duration is defined when an eye movement does not exceed 30°/s within a 40-pixel diameter region, indicating that the gaze behavior is long enough to infer that the participant was actually processing information (Olsen 2012). The percentage of valid data was calculated by dividing the total visit duration at the screen by the duration of the presentation. On average participants gazed 44.4% of the time at the jury member while presenting their story; for the ASD group this was 43.6% and for the control group 45.6%.

### Emotional Arousal

The ECG data for the heart rate assessment was collected with a Biopac MP150 Acquisition System (Biopac Systems Inc., Santa Barbara, CA) at a rate of 1000 samples/s using *Acqknowledge* software (Biopac System Inc.) throughout the experiment and analyzed afterwards using MATLAB Release 2012b (The MathWorks, Inc., Natick, Massachusetts, United States). Two Covidien Kendall Meditrace 200 ECG electrodes were placed on the chest of the participants in a II-lead configuration: below the collarbone and at the left rib of the participant. For the ECG ground measurement, a Vin-GSR electrode (Biopac 2014) was used. When a good ECG signal could not be collected due to chest hair, the electrode placed below the collarbone was moved to the right shoulder of the participant. A 2 Hz highpass filter and a 50 Hz notch filter were applied in *Acknowledge* to stabilize the ECG signal. An automated peak detection algorithm was used in MATLAB and peaks and inter-beat intervals (IBIs) were visually inspected and manually corrected by a trained researcher (RD). From the successive normal IBIs the square root of the mean squared differences (rMSSD) were calculated. This is recommended for calculating high-frequency HRV from a short-term recording with durations of several minutes (Task force of The European Society of Cardiology and the North American Society of Pacing and Electrophysiology 1996). The average HR (beats per minute; BPM) for each minute was extracted from the baseline. To control for individual differences, the minute with the lowest HR mean was determined for each participant individually. These 1-min sections served as a baseline contrast for the means during the presentation phase. For HRV, however, the baseline mean was calculated for the total baseline duration and used for comparison to the following stressful phases, as time domain methods of HRV are sensitive to the length of recording (Benevides and Lane 2015) and comparisons of variables therefore require similar durations of tasks. Given that the stressful phase lasted six minutes, the mean average of the total baseline (5 min) was used for baseline HRV.

### Emotional Awareness

A self-constructed questionnaire, based on similar questionnaires for identifying emotions that have been used before (for example by van Rijn et al. 2014), was used prompting patients to describe how they felt during the presentation. These emotions were derived from Parrott (2001) as they were considered to be relevant for the stress paradigm. Participants were asked to indicate with a slider how intensely they experienced a set of twelve different emotions (happy, frustrated, insecure, panic, enthusiasm, unpleasant, embarrassed, anxious, relieved, irritated, rejected and tense) on a continuous line of 10 cm with a scale from 0 to 100 for each of the emotions by digital query on a tablet. To assess internal validity, Cronbach’s alpha was calculated and resulted in α = 0.77, which indicates a good level of internal consistency for the questionnaire within this sample.

### Procedure

Control participants were tested at Leiden University and ASD participants were tested in Stumass residential houses. In all cases, the experiments were conducted in a quiet and stimulus-free room during day-time. The assessment consisted of a first part with neurocognitive tests and a second part with paradigms measuring arousal regulation during social situations, containing the Social Stress Task described above. First, among other cognitive tasks, the V-BD short form of the WAIS-IV was administered. After a 10-min break, the participants returned to the testing room where the electrodes were attached. After filling out a questionnaire, the autonomic arousal measurement was started and the signal quality was checked. The participant was seated 60 cm from the screen and then the eyes were calibrated with the eye tracker using a five-point calibration. The participants were instructed to sit as still as possible throughout the experiment. The instructor told the participant to look at the screen before starting the Social Stress Task. After the final phase, participants were given a questionnaire to reflect on their emotional awareness and thoughts during the presentation phase. The instructor noted the answers by digital query on a tablet. Total assessment time of the Social Stress Task was approximately 35 min. At the day of the experiment, participants received an invitation to fill out online questionnaires at home concerning demographic variables and social-cognitive functioning, among which the SRS-A questionnaire.

### Data Analysis

All analyses were conducted in IBM SPSS (v.21). Level of significance was set at *p* < 0.05. In the case of group differences, Cohen’s *d* was calculated as a measure of effect size. All data was checked for normality of the distributions using histograms and boxplots, and z-scores were calculated to check for outliers (± 3 *SD*). To measure if the groups attended to the screen and focused on the face of the jury member equally, independent t-tests with respectively visit duration to the whole screen and fixation duration at the face were conducted. For the arousal measures, group differences in baseline HR and HRV were tested with independent t-tests. In order to test whether the paradigm successfully induced stress and for testing group differences in reactivity, a repeated measures (RM) analysis of variance (ANOVA) was conducted with condition (baseline and presentation) as within-subject factor and group as between-subject factor for the two measures separately.

To test a possible group difference in emotional awareness, a multivariate ANOVA was conducted. Dependent variables were the self-reported awareness scores for all 12 emotions and group (ASD or control) was entered as the between subjects factor.

As age and total IQ differed significantly between groups, the relation with total visit duration at the screen, fixation at faces and baseline HR and HRV was analyzed by generating (nonparametric) correlations for both groups separately. No significant relations were detected. We also investigated possible relations between baseline HR and HRV and the factors that are known to influence HR(V) measurements: medication use (Kushki et al. 2014; Quintana et al. 2016; Silke et al. 2002) and sex (e.g. Sztajzel et al. 2008). No significant relations appeared for medication and sex with baseline HR and HRV and therefore none of these variables were included as covariates in further analyses.

## Results

One participant in the TD group dropped out because of technical problems (*n* = 1) and in the ASD group four participants dropped out due to technical problems (*n* = 2), incomplete assessment (*n* = 1), and physical discomfort due to the electrodes (*n* = 1). The sample characteristics for sex, age and estimated IQ for the remaining participants are given in Table 2. The groups were matched with regard to sex (*χ*^2^ (1) = .07, *p* = .51), while the ASD individuals were significantly older than the TD group (*t*(77,9) = 4.18, *p* < .001). IQ scores were all above 80, but differed between groups such that ASD participants had a higher mean score than the TD group, *p* < .001. The individuals with ASD reported to have significantly higher SRS-A total scores and subscale scores than TD individuals, p < .001. (Table 2).

**Table 2:** Group characteristics

	ASD (*N* = 47)	TD (*N* = 27)	*t*/*χ*^2^/*F*	*p*
Male sex (%)	74.0	74.1	*χ*^*2*^ = .00 (1)	.99
Age in years [*M* (*SD*)]	22.47 (2.47)	20.60 (1.52)	*t* = 4.18 (77,9)	<.001**
WAIS- IV total IQ [*M* (*SD*)]	117.82 (10.43)	107.78 (11.69)	*t* = 3.97 (78)	<.001**
SRS-A total score^a^ [*M* (*SD*)]	62.10 (9.62)	50.04 (11.23)	*F* = 23.55	<.001**

*p < .05

**p < .001

^a^T-score; missing data in the ASD group (n = 2) and the control group (n = 1)

At the beginning of the presentation phase, four participants in the ASD group dropped out because they found the phase too difficult to complete. For these participants, eye tracking and emotional awareness data were discarded and only the physiology data from the baseline phase was used for group comparisons.

### Visual Attention

Ten participants were excluded for the eye tracking data analysis to control for attention because of technical problems with the hardware (ASD; *n* = 3, TD; *n* = 7); final analyses included 44 ASD and 20 TD subjects. There were no group differences in total visit duration (*t* (62) = .32, *p* = .75) or in fixation at faces (*t* (26,87) = 1.03, *p* = .31), see Table 3.

**Table 3:** Gaze behavior: group mean (standard deviation)

	ASD (*n* = 44)	TD (*n* = 20)	*p*
Visit duration whole screen (s)	156.87 (81.43)	164.12 (89.01)	.310
Fixation duration AOI face (s)^a^	45.75 (29.60)	57.45 (46.17)	.760

^a^Data was removed for three multivariate outliers in the ASD group

For the ECG analyses, four participants were excluded: one ASD participant for all analyses; two ASD participants for HRV analysis only because they represented multivariate outliers; and one control participant for HRV analysis only because of technical problems with the hardware. Final sample numbers are provided in the figures and tables.

### Group Differences in Heart Rate

**Fig. 2:**
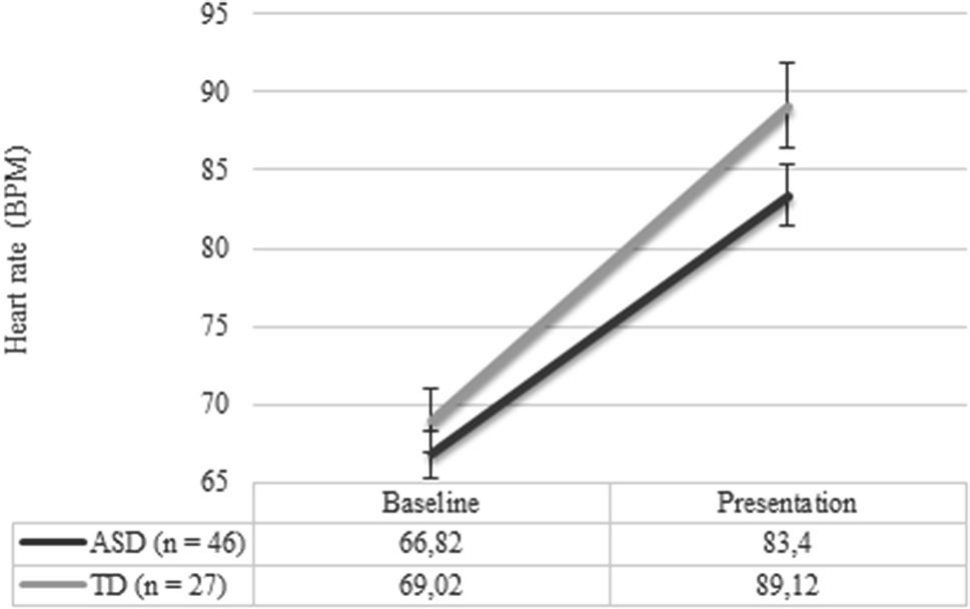
Plot of mean heart rate (HR) across the baseline and presentation phase for each group (error bars displaying SEM)

No significant differences in baseline HR were found between groups (*p* = .38), see Fig. 2. The RM ANOVA analysis to test change in HR from baseline to the stressful phases resulted in a main effect for condition (*F* (1,71) = 252.98, *p* < .001). No group by condition interaction effect (*p* = .13) was present. These results indicate that HR was increased in the stressful phases, while no group difference in HR reactivity became apparent (*p* = .14), see Fig. 2.

### Group Differences in Heart Rate Variability

**Fig. 3:**
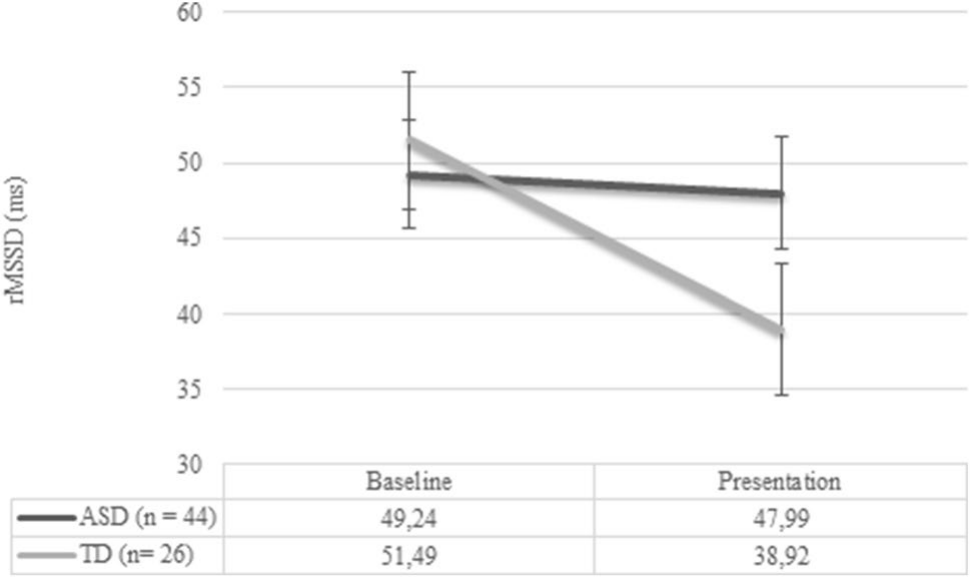
Plot of mean heart rate variability (HRV; rMSSD) across the baseline and presentation phase for each group (error bars displaying SEM)

**Fig. 4:**
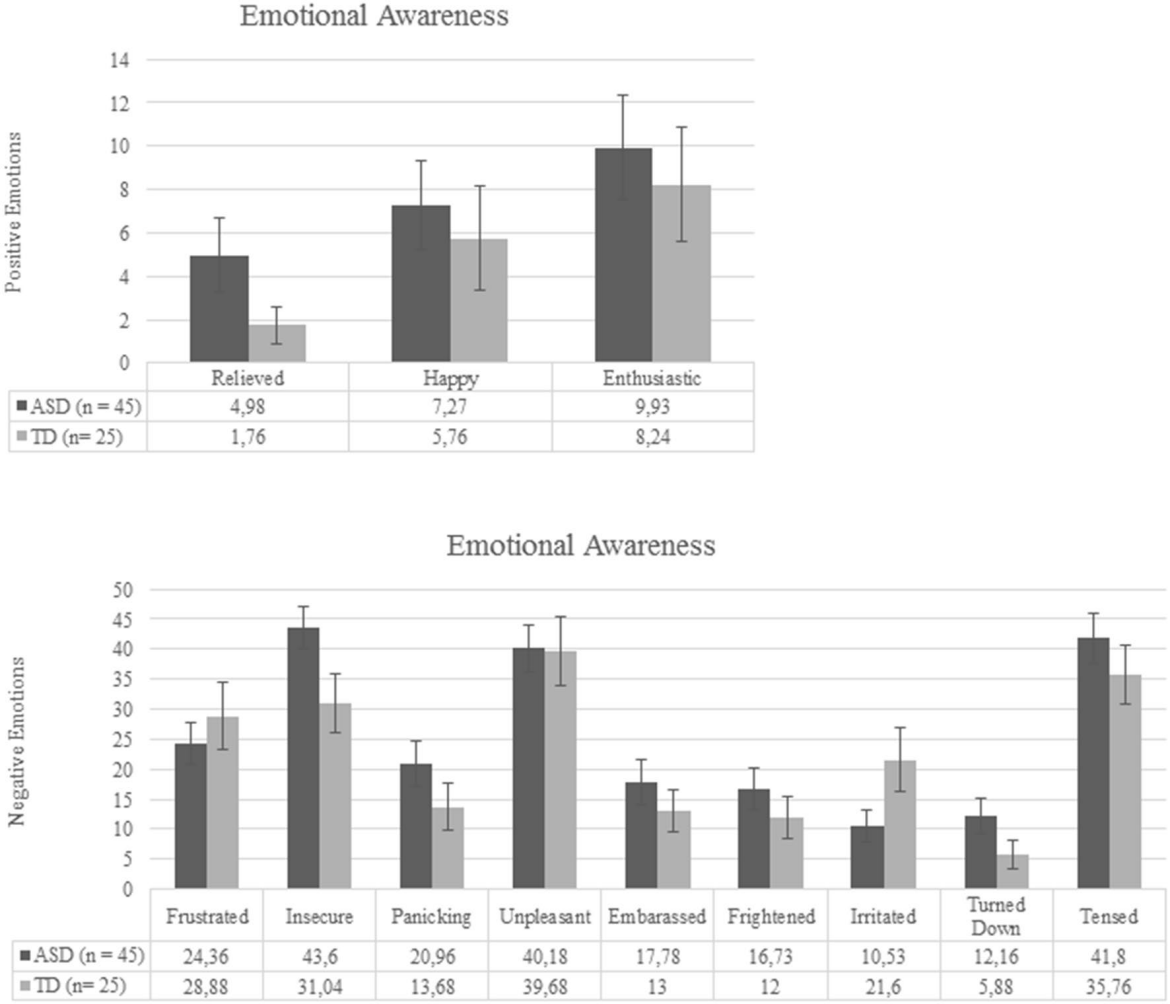
Emotional awareness during the presentation phase; group mean (error bars displaying SEM)

No significant differences in baseline HRV appeared between groups (*p* = .70), see Fig. 3. A RM ANOVA analysis to test change in HRV from baseline to the stressful phases resulted in a main effect for condition (*F* (1,68) = 8.35, *p* = .005), and a significant group-by-condition interaction effect (*F* (1,68) = 5.37, *p* = .023). The significant interaction effect indicated that the ASD individuals showed different HRV reactivity compared to the TD individuals. Figure 4 shows this difference; for the control group, HRV lowers from baseline to task, while no change can be seen in the ASD group. The effect size of the group difference in reactivity (i.e. delta score rMSSD) was *d* = 0.6 from baseline to presentation, indicating a medium effect size.

### Emotional Awareness

Three multivariate outliers (score > 3 SD from group mean on at least 2 emotions) were deleted from the dataset (*n *= 2 in the control group, and *n* = 1 in the ASD group). The MANOVA showed no significant difference in awareness of emotions between the ASD and the control group, *p* = .44. Group means per emotion can be seen in Fig. 4.

Exploratory correlational analyses showed no significant correlations between HRV and emotional awareness for the TD or ASD group. A single significant negative correlation between HR and feeling unpleasant (*r* = − .29, *p* = .047) was present in the ASD group, see supplemental table.

## Discussion

The current study aimed to address the contribution of emotional arousal regulation to social (dys) functioning in young adults with ASD, compared to their TD peers. Physiological arousal measures were included to investigate if young adults with ASD would display lower cardiac control during rest and less reactivity compared to their TD peers, as a sign of their physiological state not matching the environmental demands. Contrary to our expectations, the physiological arousal results showed comparable heart rate variability during rest and comparable heart rate reactivity to social stress between the autism and the control group. However, as hypothesized, lower HRV reactivity was found in the adults with ASD compared to their TD peers, indicating less autonomic sensitivity to the stressful social situation. Finally, no overall difference in emotional awareness was found between the ASD and the control individuals.

The decrease in HRV in TD individuals from baseline to the stressful tasks found in this study reflects a change in parasympathetic outflow and is congruent with previous research in TD individuals with the TSST (Shahrestani et al. 2015) and other psychological stress tasks like the Stroop Word Color Conflict Test (Delaney and Brodie 2000). Phasic HRV suppression is an autonomic response to stress, which reflects the withdrawal of cardiac vagal control and the activation of the defensive systems to cope with a stressor (Park and Thayer 2014; Thayer and Lane 2000). According to the Polyvagal Theory (Porges 2003), lower control of the vagal brake prevents individuals from rapidly engaging and disengaging from others and from self-soothing behaviors and calm behavioral states, thereby preventing the establishment and successful continuation of social interactions. We therefore tentatively conclude that while their ANS adequately signals social stress, young adults with ASD show maladaptive cardiac control during arousal regulation, which may play a crucial role in the difficulties in social contexts that many individuals with ASD encounter. This is in line with earlier research in adolescents with ASD by Edmiston et al. (2016), that indicated that less change in RSA from baseline during the TSST was related to greater severity of social problems in the ASD group. The authors concluded that the ASD participants showed reduced physiological self- regulation throughout the task. It is necessary to establish whether the finding of atypical HRV reactivity to socially stressful situations can be replicated in additional, sufficiently large, ASD samples. If so, then this would strengthen the notion that lower control of the vagal brake (as reflected in less HRV reactivity) constitutes a physiological marker of deviant functioning in social situations across the spectrum.

Given the finding of lower HRV reactivity in the young adults with autism, it could be speculated that one might expect higher levels of self-reported negative emotions in these individuals, as their HRV response indicates that they were not able to regulate their stress effectively. The current findings showed no overall difference in emotional awareness between the ASD and the control individuals. Based on the heart rate data, our study suggests that awareness of emotional arousal seems intact in young adults with ASD, but future research should aim to unravel the emotional consequences of diminished HRV reactivity to the social environment.

A possible explanation for often reported difficulties with emotion regulation in individuals with ASD (Laurent and Rubin 2004; Loveland 2012; Nuske et al. 2013) is the high co-occurrence of alexithymia (Bird and Cook 2013). It has been found that alexithymic individuals show low within-individual correlations among physiological, subjective, and behavioral responses (Peasley-Miklus et al. 2016; Taylor and Bagby 2004) and alexithymia has been linked to diminished vagal withdrawal (Neumann et al. 2004). Future studies investigating emotional arousal regulation in ASD should therefore incorporate measures of alexithymia, body awareness and interoception (perception of bodily changes) to disentangle the role of cardiac regulation in emotion regulation and the negative consequences this might have for social behavior in individuals with autism. Concurrently, studies with in-depth relationships between physiological arousal and self-reported emotional awareness/arousal in autism are warranted, and we recommend to incorporate for example heartbeat detection tasks and interoception questionnaires. Although exploratory analyses between physiological arousal and subjective awareness of negative emotions in the current study rendered mostly negative results, a weak but significant contradictory relationship was present in the ASD group only (higher HR associated with feeling less unpleasant), which could marginally hint at a proneness for misinterpretation of arousal signals in ASD.

It was also found in this study that both ASD and TD individuals focused very little on the face of the jury member during the stressful task (< 25% of the time), indicating high avoidance. As similar levels of avoidance were found in both groups, the observed abnormal arousal response in the individuals with autism could not be explained by differences in attention to the face and thus exposure to the stressor. Previous studies did show atypical attention towards faces in adults with ASD (Baron-Cohen et al. 2001; Fletcher-Watson et al. 2009; Riby and Hancock 2008). However, it has also been found that alexithymia predicts eye fixations in individuals with ASD (Bird et al. 2011). In the current study, the stressful situation might have influenced looking behavior in a way that maximizes avoidance, not only in the group with ASD but also in the control group. Future studies investigating simultaneous visual attention and response to stressful stimuli should therefore take into account that the nature of the stressor might influence visual attention.

This study has several limitations worth noting. First, there was a significant age difference between groups. As a result of many students with ASD experiencing study delay (Anderson et al. 2018), the mean age of the controls was lower than of the ASD group, affecting comparability. However, the age range of both groups fell well within the young adult age. Second, some researchers argue that the TSST is not a stressful task for individuals with autism (Lanni et al. 2012). However, all participants showed an increase in arousal from baseline to stressful tasks and therefore we are confident that our experimental manipulation successfully induced stress in most participants, at least on a physiological level. Furthermore, besides co-morbidities, other variables such as physical exercise and body mass index have been suggested to influence HRV (Barutcu et al. 2005; Rennie et al. 2003) but were not controlled for in this study. However, well-established factors influencing HRV, like medication use and age, were taken into consideration. Another strength of our study is that we controlled for visual attention by using eye-tracking.

In conclusion, these findings suggest reduced physiological self-regulation to psychosocial stress in young adults with ASD. Furthermore, these findings suggest that regardless of their high functioning in daily life -as reflected in high education levels and high IQ-, most of these young adults with ASD show fundamental deviations in their arousal regulation compared to their TD peers. Deficits in arousal regulation may result in being overwhelmed by emotions, which interferes with competent functioning in social situations, but may also impair the overall quality of life (Adrian et al. 2011; Mennin et al. 2007; Thompson et al. 2008). By investigating the relation of HRV with social behaviors, for example in an interactive setting, and the effect of emotion regulation training on cardiac control in ASD, it will be possible to determine whether ANS functioning is an important underlying mechanism of social behavior in autism.

## Electronic supplementary material

Below is the link to the electronic supplementary material.

Supplementary material 1 (DOCX 18 kb)
