# Supplementary material for: Emotional Arousal During Social Stress in Young Adults With Autism: Insights From Heart Rate, Heart Rate Variability and Self-Report
Source: J Autism Dev Disord. 2019 Apr 4;49(6):2524–35. doi: 10.1007/s10803-019-04000-5 (PMC6546666; doi:10.1007/s10803-019-04000-5)
Supplement: Supplementary file 1 — Supplementary material 1 (DOCX 18 kb) [file 10803_2019_4000_MOESM1_ESM.docx]

**Table 1.** *Bivariate correlations between physiology measures and emotional awareness for the ASD and the TD group*

|  | HR | | HRV | |
| --- | --- | --- | --- | --- |
|  | ASD  (*n* = 46) | TD  (*n* = 30) | ASD  (*n* = 44) | TD  (*n* = 29) |
| Frustrated  Insecure | -.25  -.21 | -.07  -.04 | -.09  -.07 | .07  .08 |
| Unpleasant  Tensed | -.30*  -.13 | -.06  .12 | -.07  -.20 | -.00  -.12 |

******p* < .05
